# The Impact of Intravenous Iron on Renal Injury and Function Markers in Patients With Chronic Kidney Disease and Iron Deficiency Without Anemia

**DOI:** 10.1016/j.ekir.2021.11.002

**Published:** 2021-11-24

**Authors:** Xenophon Kassianides, Adil Mohammad Hazara, Iain C. Macdougall, Philip A. Kalra, Sunil Bhandari

**Affiliations:** 1 Academic Renal Research Department, Hull University Teaching Hospitals NHS Trust and Hull York Medical School, Hull, UK; 2 Department of Renal Medicine, King’s College Hospital, London, UK; 3 Department of Renal Medicine, Salford Royal NHS Foundation Trust and University of Manchester, Manchester, UK

**Keywords:** chronic kidney disease, cystatin C, ferric derisomaltose, intravenous iron, iron deficiency, neutrophil gelatinase associated lipocalin

## Introduction

Intravenous (i.v.) iron is often used in the management of iron deficiency anemia in patients with nondialysis-dependent chronic kidney disease (CKD). Guidelines advocate a “high dose, low frequency approach,” which is more easily achievable in modern practice owing to new i.v. iron therapies.^1^ Concerns however persist regarding hypersensitivity and oxidative stress.^1^ These arise owing to earlier experience with older i.v. iron compounds (e.g., iron sucrose) suggesting possible nephrotoxicity owing to increased proteinuria, a phenomenon credited both directly and indirectly to oxidative damage.^2^

Newer (third generation) i.v. iron preparations (ferric carboxymaltose, ferric derisomaltose [FDI], ferumoxytol) have a more compact structure and exhibit different modes of iron release compared with their predecessors.^3^ This limits their pro-oxidant effect and potentially any resulting nephrotoxicity. As there is increasing real-world evidence on the utility and cost-effectiveness of high-dose treatment with third-generation i.v. iron preparations in populations with non–dialysis-dependent CKD,S1 it is important to explore whether any negative renal impact exists after their administration.

As part of the multicenter, double-blind, pilot, randomized, controlled “Iron & Heart” trial, markers of renal function (cystatin C and creatinine) and injury (proteinuria—glomerular damage, neutrophil-associated lipocalin [NGAL]—tubular damage) were measured. The study was primarily designed to evaluate the impact of i.v. iron (FDI) at a high dose (1000 mg) in patients with non–dialysis-dependent CKD and iron deficiency but not anemia on 6-minute walk test, as an objective measure of functional status.^4^ This was performed to establish whether similar positive effects as those exhibited in patients with heart failure exist in this population. Here, we report on the renal-associated secondary objectives evaluating the renal effect of high-dose FDI in this patient group (Supplementary Methods).

## Results

A total of 54 patients were recruited and randomized to receive FDI (*n* = 26) or placebo (*n* = 28). Baseline characteristics of the study participants are presented in Table 1. The 2 groups were well-matched with no significant differences in the majority of characteristics. Although baseline serum creatinine was significantly lower in the FDI group (158 vs. 201 μmol/L, *P* = 0.03), estimated glomerular filtration rate (eGFR) calculated using both creatinine and cystatin C were not significantly different between the 2 groups.

**Table 1:** Baseline characteristics

Characteristic	FDI	Placebo	*P* value
N	26	28	
Age, median	61	59	0.34
Sex, male	11 (44%)	15 (54%)	0.49
Ethnicity			
White	19 (73%)	23 (82%)	0.59
Black	4 (15%)	3 (11%)	
Other	3 (12%)	2 (7%)	
Smoking			
No	17 (65%)	15 (54%)	0.38
Yes—current	1 (4%)	4 (14%)	
Yes—former	8 (31%)	9 (32%)	
BMI, mean (SD)	30.768 (6.878)	30.0 1 (6.438)	0.71
Primary renal disease			
Diabetes	8 (31%)	9 (32%)	0.99
Hypertension	2 (8%)	2 (7%)	
Glomerulonephritis	7 (27%)	6 (21%)	
Adult polycystic kidney disease	2 (8%)	3 (11%)	
Reflux nephropathy	1 (4%)	2 (7%)	
Other/unknown	6 (23%)	6 (21%)	
Blood pressure, mean (SD)			
Systolic, mm Hg	138.2 (19.9)	129.4 (18.45)	0.098
Diastolic, mm Hg	78.5 (10.7)	76.2 (11.8)	0.45
Laboratory measures, median			
Creatinine, μmol/L	158 (134, 181)	201 (161, 254)	0.032^a^
eGFR-creat, ml/min per 1.73 m^2^	35 (26, 43)	26 (22, 36)	0.12
Cy C, mg/L	2.13 (1.72, 2.53)	2.28 (1.90, 2.76)	0.24
eGFR-Cy, ml/min per 1.73 m^2^	28 (21, 36)	26 (20, 31)	0.27
Hemoglobin, g/L mean (SD)	131.0 (7.4)	126.5 (11.8)	0.10
Ferritin, μg/L	55.0 (45.0, 79.0)	50.0 (26.0, 82.0)	0.37
Transferrin saturations, %	19.5 (16.0, 27.0)	19.0 (15.0, 21.0)	0.43
C-reactive protein, mg/L	4.0 (2.4, 9.5)	4.0 (2.2, 4.3)	0.32
Urinary albumin:creatinine, mg/mmol	5.9 (2.1, 48.4)	19.5 (3.3, 90.7)	0.33
NGAL, ng/ml	323.51 (252.94, 409.08)	365.29 (305.15, 430.16)	0.17

BMI, body mass index; creat, creatinine; Cy, cystatin; eGFR, estimated glomerular filtration rate; FDI, ferric derisomaltose; NGAL, neutrophil-associated lipocalin.

a *P* < 0.05

**Figure 1:**
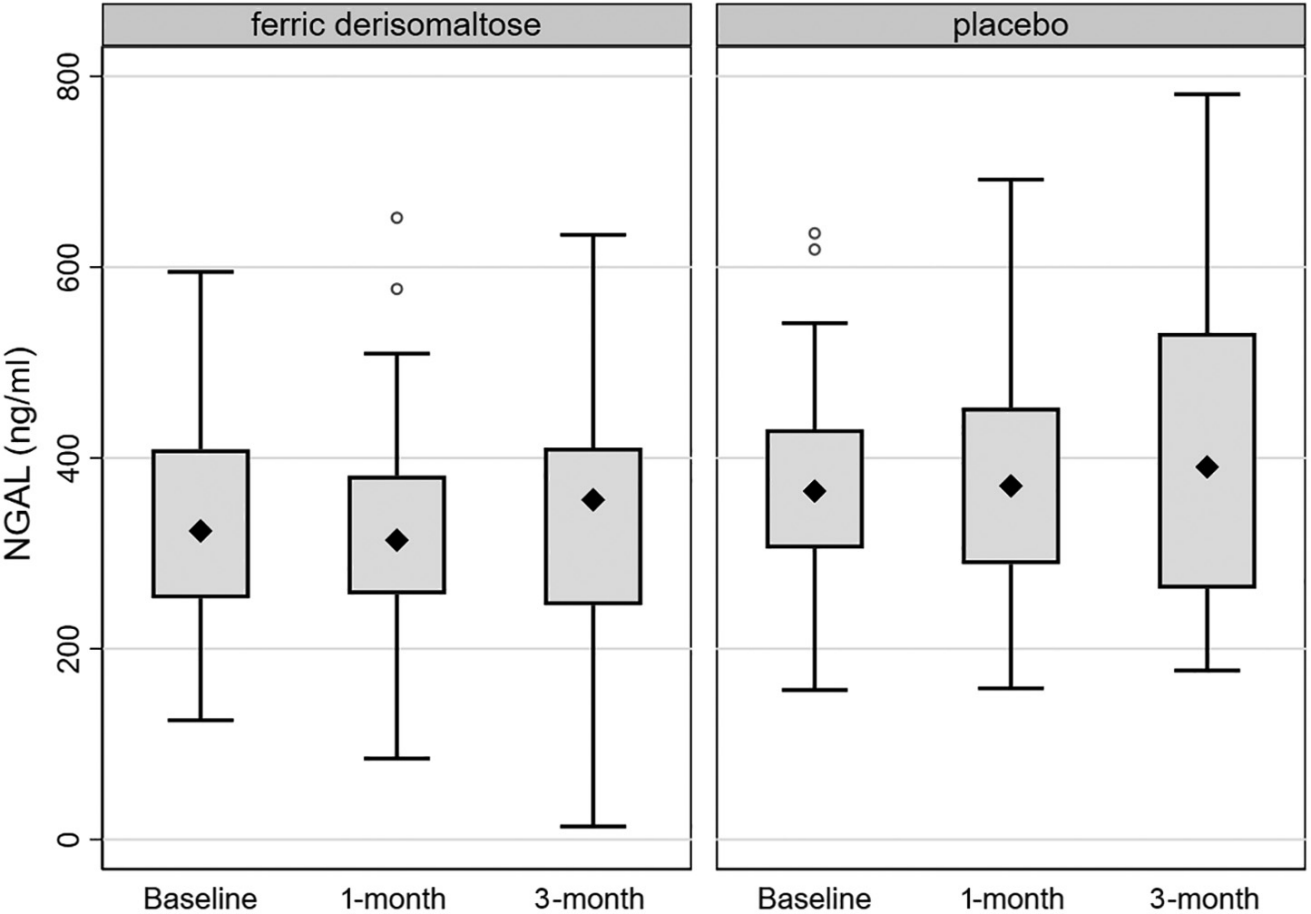
Changes in serum levels of NGAL during the follow-up period in the 2 treatment groups. *P* values nonsignificant for all pairwise comparisons between baseline versus 1-month and baseline versus 3-month values. NGAL, neutrophil-associated lipocalin.

After the FDI infusion, no significant changes were detected in the serum levels of creatinine and cystatin C nor the eGFR (Supplementary Figures S1 and S2). At baseline, the eGFR of participants in the FDI group was greater than those receiving placebo; this overall advantage was not altered throughout the study. There were no significant changes in the serum levels of NGAL in either of the treatment arms throughout the follow-up period. In the FDI group, NGAL levels at baseline, 1 month, and 3 months were 323.5, 313.9, and 356.1 ng/ml, respectively (baseline–1 month: *P* = 0.39; baseline–3 months: *P* = 0.29) and 365.2, 370.7, and 390.6 ng/ml, respectively, in the placebo group (baseline–1 month: *P* = 0.73; baseline–3 months: *P* = 0.71) (Figure 1).

**Figure 2:**
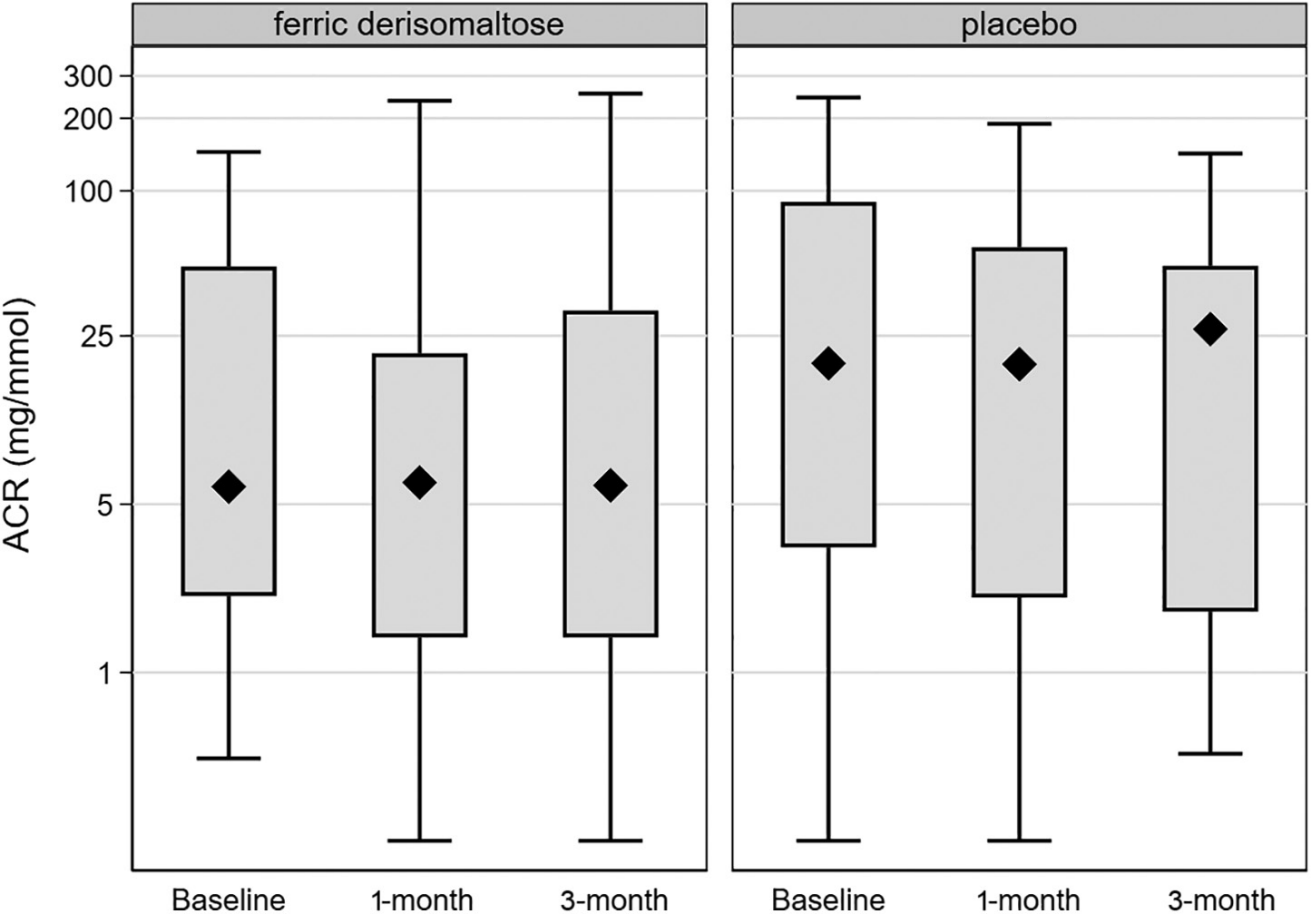
Changes in urinary albumin-to-creatinine ratio during the follow-up period in the 2 treatment groups. *P* values nonsignificant for all pairwise comparisons between baseline versus 1-month and baseline versus 3-month values. Note that the ACR results are found on a logarithmic scale. ACR, albumin-creatinine ratio.

Urinary albumin-creatinine ratio measurements were available for 22 participants in the FDI group and 24 in the placebo group. Urinary albumin-creatinine ratio levels did not change significantly post infusion in either treatment arm (Figure 2). In the FDI group, urinary albumin-creatinine ratio levels at baseline, 1 month, and 3 months were 5.9, 6.2, and 7.1 mg/mmol, respectively (baseline–1 month: *P* = 0.44; baseline–3 months: *P* = 0.28), and 19.5, 19.3, and 26.7 mg/mmol, respectively, in the placebo group (baseline–1 month: *P* = 1.00; baseline–3 months: *P* = 0.75).

## Discussion

In this study, kidney injury and function markers remained unchanged after administration of high-dose FDI at 1 and 3 months. FDI use was not associated with increases in serum levels of NGAL, a sensitive marker of acute renal tubular damage, or increases in proteinuria throughout follow-up. These findings suggest that FDI did not lead to any short term tubular or glomerular injury.

A proteinuric effect of i.v. iron has been previously displayed in patients receiving iron sucrose (100 mg) when compared with oral iron, within 15 minutes of administration.^2^ Despite a return to baseline within 24 hours, that study concluded that certain i.v. iron compounds could be associated with nephrotoxicity. In this study, with FDI, there was no increase in proteinuria level at any follow-up point compared with placebo. In addition, there was no statistically significant change in NGAL with FDI treatment, similar to the IRON-TURTLE trial using a dose of 1000 mg of ferric carboxymaltose in patients with heart failure and CKD and correlating with *in vivo* and *in vitro* evidence relevant to third-generation i.v. iron compounds.^5^^,^^6^ Indeed, such evidence has indicated that third-generation i.v. iron compounds seem to be “bioneutral” in terms of renal injury and oxidative stress when compared with earlier preparations.S2

In this study, no detriment in terms of creatinine or cystatin C (and their derived eGFR) was observed with FDI compared with placebo. This is in agreement with previous studies with non–dialysis CKD participants.^5^^,^^7^^,^^8^ The REVOKE trial (*n* = 136, non–dialysis-dependent CKD with iron deficiency anemia) compared i.v. iron sucrose with oral iron in terms of measured glomerular filtration rate using iothalamate clearance in a 2-year period.^7^ Measured glomerular filtration rate declined similarly irrespective of treatment modality and after additional demographic, medication, and comorbidity adjustments.^7^ During the FIND-CKD trial, 626 non–dialysis-dependent CKD patients were randomized to oral iron or i.v. ferric carboxymaltose and similarly demonstrated no difference in eGFR between the 2 treatment modalities in a 12-month period.^8^ In patients with CKD and reduced ejection fraction, administration of high dose ferric carboxymaltose also did not produce any impact on cystatin C or other markers of renal function.^5^ It is noteworthy that an analysis of the FAIR-HF trial evaluating the impact of ferric carboxymaltose in patients with established systolic heart failure concluded that correction of iron deficiency with i.v. ferric carboxymaltose led to a statistically significant improvement in eGFR when compared with placebo.^9^

The neutral results of this study on renal injury may be caused by a combination of factors potentially associated with the iron preparation used. Intriguingly, alleviation of iron deficiency may be renoprotective with recent evidence suggesting correction of iron deficiency in murine uremic models promotes an overall antioxidant effect, whereas the notion of renal preconditioning through activation of the NRFT2 pathway is also explored.S3^,^S4

Several limitations are present in this study, and the results therefore need to be treated with caution. The study did not focus on an ultra-acute effect of i.v. iron as that previously displayed in literature. This study focused on short- to medium-term renal damage after i.v. iron administration, and it is therefore not possible to draw any conclusions on a potential cumulative effect of repeated iron administrations. In addition, as these are secondary outcomes, the study is underpowered to detect true statistical significance.

In conclusion, the results from the Iron and Heart trial with FDI treatment add further to the safety evidence surrounding the use of i.v. iron in patients with non–dialysis-dependent CKD, especially in terms of renal injury and function. Further markers of nephrotoxicity can be incorporated in such research (e.g., urinary NGAL, beta-2-microglobulin) to investigate further possible renal injury. These results are reassuring with regard to the safety of conducting further large-scale trials in the treatment of iron-deficient patients with CKD but not anemia to identify any impact on quality of life and functional status, similar to that already found in patients with heart failure.

## Disclosure

SB, ICM, and PAK have received honoraria for lectures, attended expert opinion committees and advisory boards, and received educational funds to attend international nephrology meetings from Pharmacosmos A/S and Vifor Pharma. XK and AMH declared no competing interests.

## Supplementary Material

**Supplementary Methods**

**Supplementary References**

**Figure S1.** Changes in serum creatinine and cystatin C in the FDI and placebo groups.

**Figure S2.** Changes in eGFR in the FDI and placebo groups.

**STROBE Statement** (PDF)
