# Supplementary material for: The Impact of Intravenous Iron on Renal Injury and Function Markers in Patients With Chronic Kidney Disease and Iron Deficiency Without Anemia
Source: Kidney Int Rep. 2021 Nov 24;7(2):322–6. doi: 10.1016/j.ekir.2021.11.002 (PMC8820978; doi:10.1016/j.ekir.2021.11.002)
Supplement: Supplementary File (PDF) [file mmc1.pdf]

Supplementary methods:

The 'Iron & Heart' trial (EudraCT No. 2014-004133-6) was a double-blinded multicenter randomized controlled study investigating the impact of iron on non-anemic (Hemoglobin ≥110 g/L up to and including 150 g/L) but iron deficient (Serum ferritin level <100 μg/L and/or transferrin saturation ≤20%) individuals with non-dialysis dependent CKD. The protocol and inclusion/exclusion criteria have previously been published but, in brief, participants, following informed consent, were randomized using online software in a 1:1 fashion, to receive either 1000 mg of FDI or placebo (100 ml of 0.9% NaCl) and were monitored at 1 and 3 month intervals. Blood tests and other study-related procedures were performed at baseline and at each follow-up appointment. The study was conducted according to and adhering to Good Clinical Practice guidelines and the Declaration of Helsinki subsequent to approval by the Research Ethics Committee Yorkshire and The Humber – Leeds East, UK (REC no. 14/YH/1209).

Renal function was assessed using available biomarkers. Serum creatinine was enzymatically assessed using the AU5800 analyzer (Beckman Coulter, Nyon, Switzerland). Cystatin C was analyzed using the Gentian Cystatin C immunoassay (Gentian Diagnostics AS, Moss, Norway), and eGFR creatinine ($eGFR_{creat}$) and cystatin C ($eGFR_{cystatinC}$) was calculated using the CKD-EPI equation [20]. Proteinuria was measured on spot urine samples and expressed as urine protein:creatinine and albumin:creatinine ratios (ACR). In two of the participating centers, urinary protein:creatinine ratio (PCR) was used as a measure of urinary protein loss. PCR values were converted to ACR using a validated method first described by Weaver *et al* [S5]. Cystatin C was analyzed using the Gentian cystatin C immunoassay (Gentian Diagnostics AS, Moss, Norway). An enzyme-linked immunosorbent assay (ELISA Human NGAL Kit –

LifeTechnologies | Thermo Fisher Scientific, Carlsbad, CA, USA) was used to analyze NGAL.

Baseline participant demographics, clinical and laboratory characteristics were summarized using means, medians and proportions as appropriate. Differences between FDI and placebo groups were explored using two-sample t-test, Wilcoxon rank-sum test or Pearson's chi squared test as appropriate. Measurements taken at baseline and the follow-up intervals were compared using Wilcoxon matched-pairs signed-rank test at each time point as the variables did not follow normal distribution. A p value of < 0.05 was considered to be statistically significant. Statistical analyses were performed using Stata/IC 16 (StataCorp LLC, Texas, USA).

Supplementary references:

S1:Kalra PA, Bhandari S, Spyridon M, et al. NIMO-CKD-UK: a real-world, observational study of iron isomaltoside in patients with iron deficiency anaemia and chronic kidney disease. *BMC Nephrology*. 2020;21(1):1-10. doi:10.1186/s12882-020-02180-2

S2: Kassianides X, Gordon A, Sturmey R, Bhandari S. The comparative effects of intravenous iron on oxidative stress and inflammation in patients with chronic kidney disease and iron deficiency: a randomized controlled pilot study. *Kidney Research and Clinical Practice*. 2021;40(1):89-98. doi:10.23876/j.krcp.20.120

S3: Nuhu F, Seymour AM, Bhandari S. Impact of intravenous iron on oxidative stress and mitochondrial function in experimental chronic kidney disease. *Antioxidants*. 2019;8(10):498. doi:10.3390/antiox8100498

S4: Zager RA, Johnson ACM, Therapeutics R. Iron sucrose ('RBT-3') activates the hepatic and renal HAMP1 gene, evoking renal hepcidin loading and resistance to cisplatin nephrotoxicity. *Nephrology Dialysis Transplantation*. 2021;36(3):465-474. doi:10.1093/ndt/gfaa348

S5: Weaver RG, James MT, Ravani P, et al. Estimating urine albumin-to-creatinine ratio from protein-to-creatinine ratio: Development of Equations using Same-Day Measurements. *Journal of the American Society of Nephrology*. 2020;31(3):591-601. doi:10.1681/ASN.2019060605

Supplementary figures:

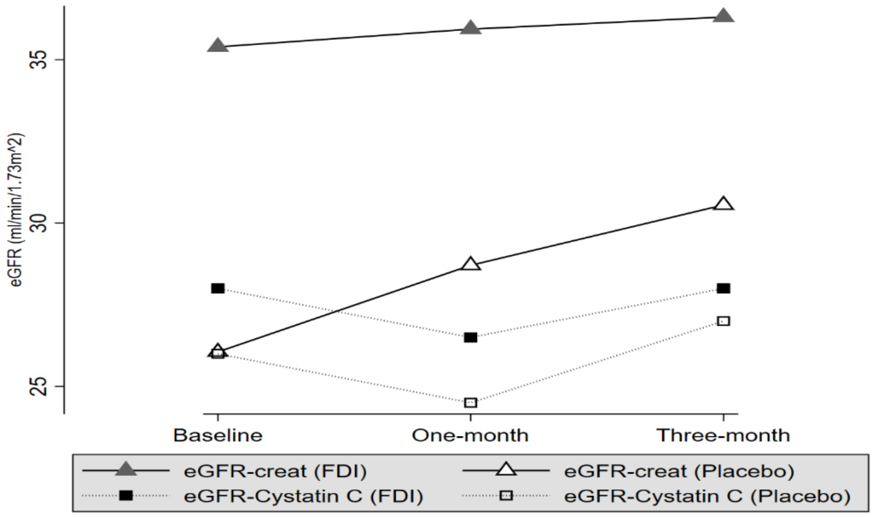

Figure 1. Changes in Serum creatinine and Cystatin C in the FDI and placebo groups. *P* values non-significant for all pairwise comparisons between baseline vs 1-month and baseline vs 3-month values.

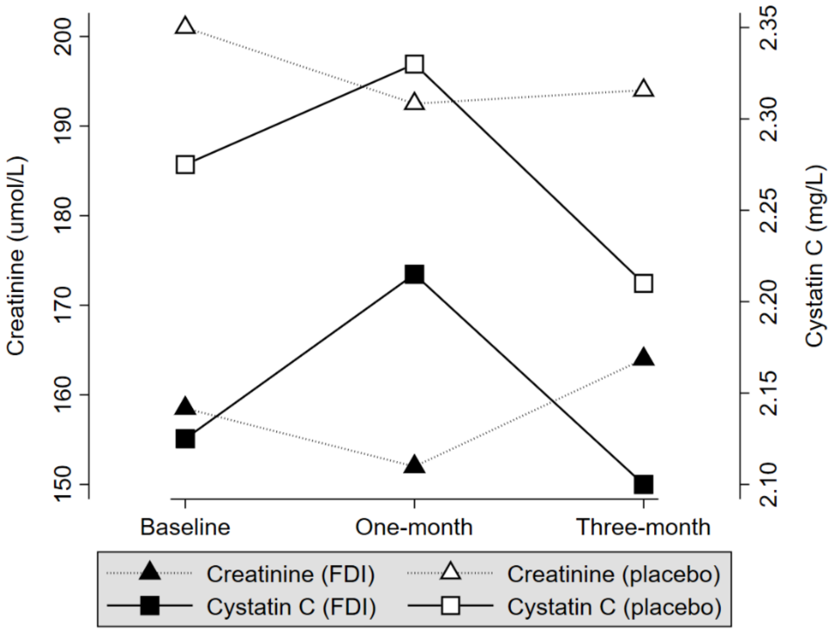

Figure 2. Changes in $eGFR_{creat}$ and $eGFR_{CystatinC}$ in the FDI and placebo groups. *P* values non-significant for all pairwise comparisons between baseline vs 1-month and baseline vs 3-month values.
